# The effects of aerobic exercise on 24-hour mean blood glucose levels measured by continuous glucose monitoring in type 2 diabetes: a meta-analysis

**DOI:** 10.3389/fphys.2024.1496271

**Published:** 2024-12-23

**Authors:** Chou Wang, Shaokai Tang

**Affiliations:** ^1^ School of Sport, Health and Exercise, Loughborough University, Loughborough, United Kingdom; ^2^ College of Sports Science, Jishou University, Jishou, China

**Keywords:** meta-analysis, T2DM, aerobic exercise, 24-hour mean glucose, CGM

## Abstract

**Purpose:**

To examine the effects of structured aerobic exercise on 24-hour mean blood glucose outcomes assessed by continuous glucose monitors in adults with type 2 diabetes.

**Methods:**

The study established specific inclusion and exclusion criteria and conducted a comprehensive search across five databases, including PubMed, Web of Science, Embase, Cochrane Library, and EBSCOhost from the start year of each database’s coverage to 22 July 2024. The quality of the included studies was evaluated using the Cochrane Handbook 5.1 guidelines. Data analysis was performed using Review Manager 5.4 to determine effect sizes, conduct sensitivity analyses, assess potential biases, and perform subgroup analyses.

**Results:**

A total of 1,034 articles were retrieved, and after 4 rounds of screening, 13 articles were finally selected for meta-analysis. The study included 626 participants (30% female; mean ± SD: age, 59.4 ± 6.4 years; BMI, 29.61 ± 2.24 kg/m^2^), including 330 in the experimental group and 296 in the control group. The results of the meta-analysis showed that aerobic exercise can improve the 24-hour mean blood glucose in patients with T2DM (d = −0.65, 95% CI: −0.75 to −0.55, *p* < 0.05). Subgroup analysis showed that moderate-intensity and high-intensity aerobic exercise can improve the 24-hour mean blood glucose in patients with T2DM (d = −0.71, 95% CI: −0.81 to −0.60, *p* < 0.05), (d = −0.60, 95% CI: −0.98 to −0.22, *p* < 0.05). Also, 20–40 min and 40–60 min of aerobic exercise per session can improve the 24-hour average blood glucose in patients with T2DM (d = −0.75, 95% CI: −0.91 to −0.59, *p* < 0.05), (d = −0.59, 95% CI: −0.71 to −0.46, *p* < 0.05). Aerobic exercise can improve the 24-hour mean blood glucose in patients with T2DM who have a body mass index (BMI) between 29 and 30 kg/m^2^, as well as those with a BMI greater than 30 kg/m^2^ (d = −0.65, 95% CI: −0.94 to −0.36, *p* < 0.05), (d = −0.76, 95% CI: −0.87 to −0.64, *p* < 0.05).

**Conclusion:**

Aerobic exercise can improve the 24-hour mean blood glucose in patients with T2DM. Additionally, 20–60 min of aerobic exercise with moderate intensity, and high intensity can improve the 24-hour mean blood glucose in patients with T2DM who have a BMI greater than 29 kg/m^2^.

**Systematic Review Registration:**

https://www.crd.york.ac.uk/prospero/, identifer PROSPERO CRD42024590812

## 1 Introduction

Diabetes has become a global health problem. By 2030, it is projected that 11.3% of the global population will have diabetes (Zhang et al., 2023). Complications arising from diabetes, including cardiovascular disease, kidney disease, and retinopathy, are closely related to blood glucose fluctuations (Sartore et al., 2023). Maintaining stable blood sugar levels throughout the day is crucial for the long-term health of patients. The 24-hour mean blood glucose is the average of blood glucose levels measured over a 24-hour period, often obtained using continuous glucose monitor (CGM) devices. These devices can measure blood glucose levels every 5 min, and long-term use allows patients to collect a large amount of blood glucose data over 24 h (Edelman et al., 2018). Compared to other diagnostic indicators for diabetes, CGM devices offer advantages such as large-scale data collection, convenient operation, and real-time monitoring.

Dietary, pharmacological, and exercise interventions are common methods to control blood glucose levels in patients with type 2 diabetes mellitus (T2DM) (Zhang et al., 2023). T2DM occurs due to the body’s inability to use insulin effectively and accounts for 90% of diabetes cases globally (Wu et al., 2020). Dietary interventions often require strict control of food types and calorie intake, which can be challenging to maintain long-term. Pharmacological interventions require regular medication use, which can lead to dependency and side effects (Remelli et al., 2022). Exercise interventions, however, are flexible, convenient, and can be easily incorporated into daily routines. Resistance exercises help strengthen muscles, but they pose a risk of injury to beginners and older adults. Taijiquan, a traditional Chinese mind-body exercises, is beneficial for balance, cardiovascular and respiratory function (Li et al., 2024). However, it is complex and requires time to learn and master. Aerobic exercise, consistently ranked among the top 20 global fitness trends in the 2024 ACSM Worldwide Fitness Trends report, is one of the most widely recommended safe and highly efficient interventions for enhancing cardiometabolic health (A’Naja et al., 2024; Batrakoulis et al., 2022a). Among them, high intensity interval training (HIIT), is especially highly efficient in improving physical health, including cardiorespiratory fitness, and psychological health, such as increased quality of life and enjoyment in exercise (Batrakoulis et al., 2021; Batrakoulis and Fatouros, 2022). These physical and psychological benefits brought by aerobic exercise make it a ideal intervention for managing chronic conditions such as T2DM.

Current studies present conflicting opinions regarding the effect of aerobic exercise on 24-hour average blood glucose. Oberlin et al. (2014) reported that a single 60-minute aerobic exercise session can significantly improve the 24-hour mean blood glucose in patients with T2DM, from 6.62 to 5.98 mmol/L. Additionally, the area under the blood glucose curve after lunch was markedly reduced following exercise compared to the control group. In contrast, Rees et al. (2019) found while a single session of fast walking significantly decreased blood glucose levels during the exercise by 1.56 mmol/L in middle-aged and elderly T2DM patients, it did not significantly change the 24-hour average blood glucose, fasting blood glucose (FBG), postprandial blood glucose, or blood glucose variability compared to the control. These findings suggest that variations in exercise type, duration, and intensity may account for the differences in 24-hour average blood glucose results observed in aerobic exercise studies involving patients with T2DM.

Based on this, a meta-analysis was conducted to assess the effect of aerobic exercise on 24-hour mean blood glucose levels measured using CGM in patients with T2DM. This analysis aims to clarify the effects of different exercise periods, intensities, frequencies, and duration on the 24-hour mean blood glucose of T2DM patients with different BMIs. The findings will provide valuable insights and references for future research on blood glucose management in patients with T2DM.

## 2 Method

### 2.1 Search strategy

Searches were conducted in PubMed, Web of Science, Embase, Cochrane Library, and EBSCOhost from the start year of each database’s coverage to 22 July 2024. The search strategy employed a combination of subject terms and free terms. The search terms are presented in Table 1. The search terms were divided into three groups based on intervention method, research subject, and outcome indicator, with terms within each group connected by OR. The groups are then combined using AND. After the literature search, two researchers independently screened the articles in a double blend manner. The search results were imported into EndNote 21 literature management software, where duplicate records were identified and removed by reviewing the titles and abstracts. Subsequently, the full texts were downloaded and reviewed for data extraction. This process was also conducted independently by two researchers. In cases of disagreement, a third researcher was consulted to resolve whether to include the study.

**TABLE 1:** Search terms and search formulas.

Search term classification	Search term
Intervention method	Exercise [Mesh] or Exercises or Exercise, Physical or Exercises, Physical or Physical Exercise or Physical Exercises or Physical Activity or Activities, Physical or Activity, Physical or Physical Activities or Exercise, Aerobic or Aerobic Exercise or Aerobic Exercises or Exercises, Aerobic or Exercise, Isometric or Exercises, Isometric or Isometric Exercises or Isometric Exercise or Acute Exercise or Acute Exercises or Exercise, Acute or Exercises, Acute or Exercise Training or Exercise Trainings or Training, Exercise or Trainings, Exercise
Research subject	Diabetes Mellitus, Type 2 [Mesh] or Diabetes Mellitus, Adult-Onset or Adult-Onset Diabetes Mellitus or Diabetes Mellitus, Adult Onset or Diabetes Mellitus, Ketosis-Resistant or Diabetes Mellitus, Ketosis Resistant or Ketosis-Resistant Diabetes Mellitus or Diabetes Mellitus, Non Insulin Dependent or Diabetes Mellitus, Non-Insulin-Dependent or Non-Insulin-Dependent Diabetes Mellitus or Diabetes Mellitus, Stable or Stable Diabetes Mellitus or Diabetes Mellitus, Type II or NIDDM or Diabetes Mellitus, Noninsulin Dependent or Diabetes Mellitus, Maturity-Onset or Diabetes Mellitus, Maturity Onset or Maturity-Onset Diabetes Mellitus or Maturity Onset Diabetes Mellitus or MODY or Diabetes Mellitus, Slow-Onset or Diabetes Mellitus, Slow Onset or Slow-Onset Diabetes Mellitus or Type 2 Diabetes Mellitus or Noninsulin-Dependent Diabetes Mellitus or Noninsulin Dependent Diabetes Mellitus or Maturity-Onset Diabetes or Diabetes, Maturity-Onset or Maturity Onset Diabetes or Type 2 Diabetes or Diabetes, Type 2 or Diabetes Mellitus, Noninsulin-Dependent
Outcome indicator	Continuous Glucose Monitoring [Mesh] or Glucose Monitoring, Continuous or Monitoring, Continuous Glucose or Monitorings, Continuous Glucose or Continuous Glucose Monitoring Device or CGM Device or CGM Devices or Device, CGM or Devices, CGM
Research method	RCT

### 2.2 Inclusion and exclusion criteria

#### 2.2.1 Inclusion criteria

Study participants: Eligible participants were adult patients with T2DM, aged 18 years or older.

Study intervention: Studies were included if they clearly defined the exercise period, intensity, frequency, and duration of structured exercise and reported the effects of these parameters. Articles that merely encouraged participants to become more active or participate in sports without providing a substantially structured exercise program or exercise monitoring (e.g., direct supervision or a logbook) were excluded.

Research comparison: A non-exercise control condition was required for comparison with the exercise conditions. Both randomized and non-randomized comparisons (e.g., before-and-after studies) were eligible, as were trials employing a parallel or cross-over design. However, studies comparing the combined use of exercise and dietary interventions with a control condition that did not receive a dietary intervention were deemed ineligible.

Measurement results: The study required data collected using CGM devices over a 24-hour period under both exercise and control conditions. The primary outcome measure was the mean 24-hour blood glucose level, while the secondary outcome measure was glycosylated hemoglobin (HbA1c).

### 2.3 Data extraction

Two researchers independently extracted relevant data from the articles:

1. Basic article data, including the first author and the year of publication.
2. Participant data, including age, gender, subject population, sample size, and BMI.
3. Exercise intervention details, including exercise period, exercise intensity, exercise frequency, and exercise duration.
4. Outcome indicators, including 24-hour mean blood glucose measured by CGM and HbA1c.

### 2.4 Quality evaluation

The quality of the included studies was assessed using Review Manager 5.4, following the guidelines of the Cochrane Handbook version 5.1. The assessment criteria included random allocation methods, allocation concealment, blinding, data outcome integrity, selective reporting, and other potential sources of bias. Studies were classified as low risk if they complied with these standards, high risk if they did not comply, and medium risk if there was no information provided, with the reason for this classification noted. Two researchers conducted the quality evaluation together. In the event of a disagreement, a third researcher was involved in a joint discussion to reach a consensus.

### 2.5 Data analysis

Review Manager 5.4 was used to pool effect sizes and assess publication bias. The outcome indicators included in the study were continuous variables, so the effect sizes were expressed as the mean ± standard deviation. Heterogeneity between groups was measured using the I^2^ statistic. If I^2^ was less than 50%, indicating no significant statistical heterogeneity, a fixed-effect model was used for the analysis. If I^2^ was 50% or higher, a random-effects model was applied. Funnel plots were used to evaluate the risk of bias, and sensitivity analyses were conducted to assess the robustness of the results.

## 3 Results

### 3.1 Search results

**FIGURE 1:**
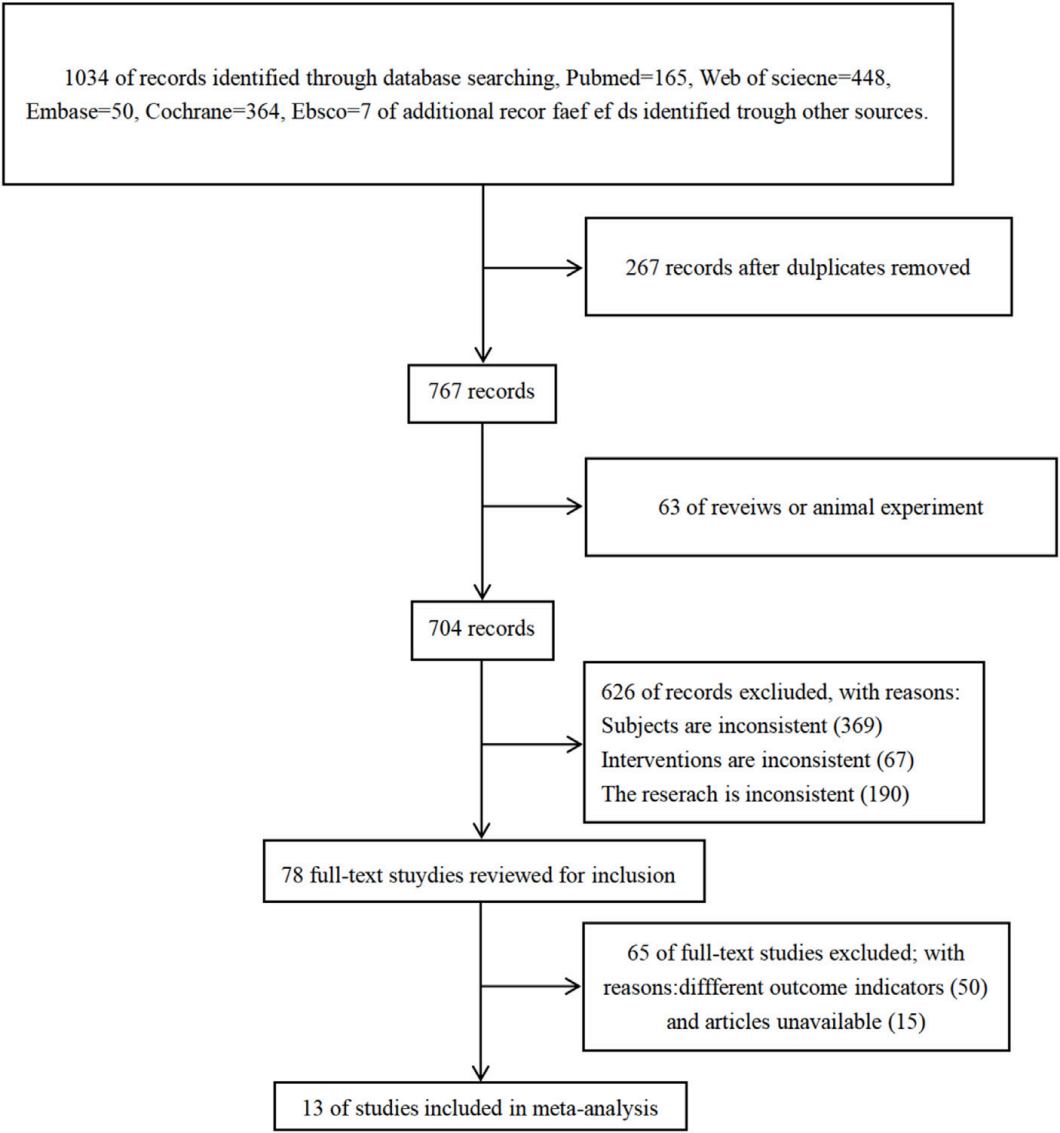
Flow chart for literature screening.

A total of 1.034 articles were retrieved: 165 from the PubMed database, 448 from the Web of Science database, 50 from the Embase database, 364 from the Cochrane database, and 7 from the EBSCO database. After reviewing the titles, abstracts, and full texts, 1,021 articles were excluded. Ultimately, 13 articles were selected for the meta-analysis. The screening process is illustrated in the figure below (see Figure 1).

### 3.2 Inclusion of the basic characteristics of the article

Study participants: A total of 626 adult participants with T2DM (30% female) were included in the study. Their age was 59.4 ± 6.4 years, and their BMI was 29.61 ± 2.24 kg/m^2^. Exercise duration includes shorter durations associated with transient blood glucose changes, involving 1–2 days, and longer durations promoting adaptive blood glucose changes, ranging from 1 to 4 months. The frequency of exercise can range from once to seven times a week. The intensity range included peak power: 50%–95%; maximum power: 35%–70%; maximum heart rate: 65%–94%; heart rate reserve: 50%–75%; maximum oxygen uptake: 50%–93%; maximum load capacity: 50%; ventilatory threshold: 85%; maximum energy expenditure rate: 55%–70%. Exercise time ranged from 10 min to 60 min (see Table 2).

**TABLE 2:** Article information extraction table.

Source of literature	Subject population	Total sample size	Gender	Age	Exercise period	Exercise frequency (times/weeks)	Exercise intensity	Exercise Time (mins)	BMI (kg/m^2^)
Winding et al. (2018)	T2DM patients	19	Male, Female	58 ± 8	11 weeks	3	50% of Wpeak	40	28.0 ± 3.5
Winding et al. (2018)	T2DM patients	20	Male, Female	54 ± 6	11 weeks	3	95% Wpeak	20	28.0 ± 3.5
Karstoft et al. (2013)	T2DM patients	20	Male, Female	60.8 ± 2.2	4 months	5	55% of the peak energy-expenditure rate	60	29.7 ± 1.9
Karstoft et al. (2013)	T2DM patients	20	Male, Female	57.5 ± 2.4	4 months	5	70% of the peak energy-expenditure rate	60	29.7 ± 1.9
Yang et al. (2024)	T2DM patients	44	Male, Female	40.5 ± 4.78	3 months	3	50%–75% VO2 max	30–60	25.48 ± 3.75
Marcotte-Chénard et al. (2024)	T2DM patients	28	Female	69.9 ± 4.3	One day	1	90% HR max	38	33.2 ± 5.6
Marcotte-Chénard et al. (2024)	T2DM patients	28	Female	69.9 ± 4.4	One day	1	90% of HRmax	35	33.2 ± 5.6
Munan et al. (2020)	T2DM patients	25	Male, Female	65 ± 9.0	12 days	7	99 ± 18 heart rate (beats/min)	50	27.2 ± 3.5
Munan et al. (2020)	T2DM patients	26	Male, Female	65 ± 9.0	12 days	7	106 ± 17 heart rate (beats/min)	50	27.2 ± 3.5
Munan et al. (2020)	T2DM patients	25	Male, Female	65 ± 9.0	12 days	7	106 ± 19 heart rate (beats/min)	50	27.2 ± 3.5
Rees et al. (2019)	T2DM patients	63	Male, Female	64 ± 8	One day	1	65% ± 9% of HRmax	50	30.5 ± 6.5
Metcalfe et al. (2018)	T2DM patients	22	Male	52 ± 6	One day	1	50% of Wmax	30	29.1 ± 3.7
Metcalfe et al. (2018)	T2DM patients	22	Male	52 ± 6	One day	1	90HRmax	25	29.1 ± 3.7
Metcalfe et al. (2018)	T2DM patients	22	Male	52 ± 6	One day	1	91% ± 3% of HRmax	10	29.1 ± 3.7
Karstoft et al. (2017)	T2DM patients	28	Male, Female	65 ± 2	2 weeks	5	75.6% ± 2.5% of VO2peak	60	>18 kg/m^2^ but <40 kg/m^2^
Karstoft et al. (2017)	T2DM patients	28	Male, Female	65 ± 2	2 weeks	5	90.0% ± 3.6% ˙VO2peak	60	>18 kg/m^2^ but <40 kg/m^2^
Myette-Côté et al. (2016)	T2DM patients	20	Male, Female	59.0 ± 9.6	One day	1	85% of VT (Ventilatory Threshold)	50	29.5 ± 4.7
Haxhi et al. (2016)	T2DM patients	18	Male	58.2 ± 6.6	One day	1	50% of heart rate reserve	40	30.2 ± 3.1
Haxhi et al. (2016)	T2DM patients	18	Male	58.2 ± 6.6	One day	1	50% of heart rate reserve	20	30.2 ± 3.1
Oberlin et al. (2014)	T2DM patients	18	Male, Female	60.3 ± 1.0	One day	1	60%–75% of heart rate reserve	60	36.0 ± 1.1
van Dijk et al. (2012)	T2DM patients	60	Male	60 ± 1	2 days	1	50% maximal workload capacity	60	30.4 ± 0.7
van Dijk et al. (2012)	T2DM patients	60	Male	60 ± 1	One day	2	50% maximal workload capacity	30	30.4 ± 0.7
Manders et al. (2010)	T2DM patients	18	Male	57.0 ± 2.0	One day	1	35% Wmax	60	29.0 ± 1.0
Manders et al. (2010)	T2DM patients	18	Male	57.0 ± 2.0	One day	1	70% Wmax	30	29.0 ± 1.0

### 3.3 Assessment of study quality

**FIGURE 2:**
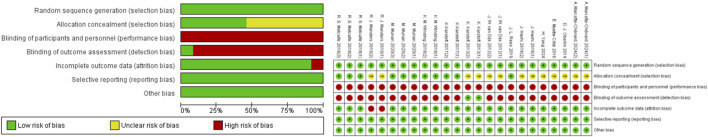
Quality evaluation diagram for literature.

The quality of the included studies was evaluated, and all studies met the random sequence generation; 13 studies met the criteria for randomization (Oberlin et al., 2014; Rees et al., 2019; Winding et al., 2018; Karstoft et al., 2013; Yang et al., 2024; Marcotte-Chénard et al., 2024; Munan et al., 2020; Metcalfe et al., 2018; Karstoft et al., 2017; Myette-Côté et al., 2016; Haxhi et al., 2016; van Dijk et al., 2012; Manders et al., 2010); and 5 articles met the allocation concealment requirement (Rees et al., 2019; Winding et al., 2018; Munan et al., 2020; Metcalfe et al., 2018; Karstoft et al., 2017). None of the included articles met the criterion for blinding of participants and personnel. One article met the requirement for blinding of outcome assessment (Karstoft et al., 2013). Furthermore, 12 articles met the criterion for incomplete outcome data (Oberlin et al., 2014; Rees et al., 2019; Winding et al., 2018; Karstoft et al., 2013; Yang et al., 2024; Marcotte-Chénard et al., 2024; Munan et al., 2020; Metcalfe et al., 2018; Karstoft et al., 2017; Myette-Côté et al., 2016; Haxhi et al., 2016; van Dijk et al., 2012); 13 articles met the criterion for selective reporting (Oberlin et al., 2014; Rees et al., 2019; Winding et al., 2018; Karstoft et al., 2013; Yang et al., 2024; Marcotte-Chénard et al., 2024; Munan et al., 2020; Metcalfe et al., 2018; Karstoft et al., 2017; Myette-Côté et al., 2016; Haxhi et al., 2016; van Dijk et al., 2012; Manders et al., 2010); and 12 articles met the criterion for other sources of bias (Oberlin et al., 2014; Winding et al., 2018; Karstoft et al., 2013; Yang et al., 2024; Marcotte-Chénard et al., 2024; Munan et al., 2020; Metcalfe et al., 2018; Karstoft et al., 2017; Myette-Côté et al., 2016; Haxhi et al., 2016; van Dijk et al., 2012; Manders et al., 2010) (Figure 2).

### 3.4 Effect size evaluation

**FIGURE 3:**
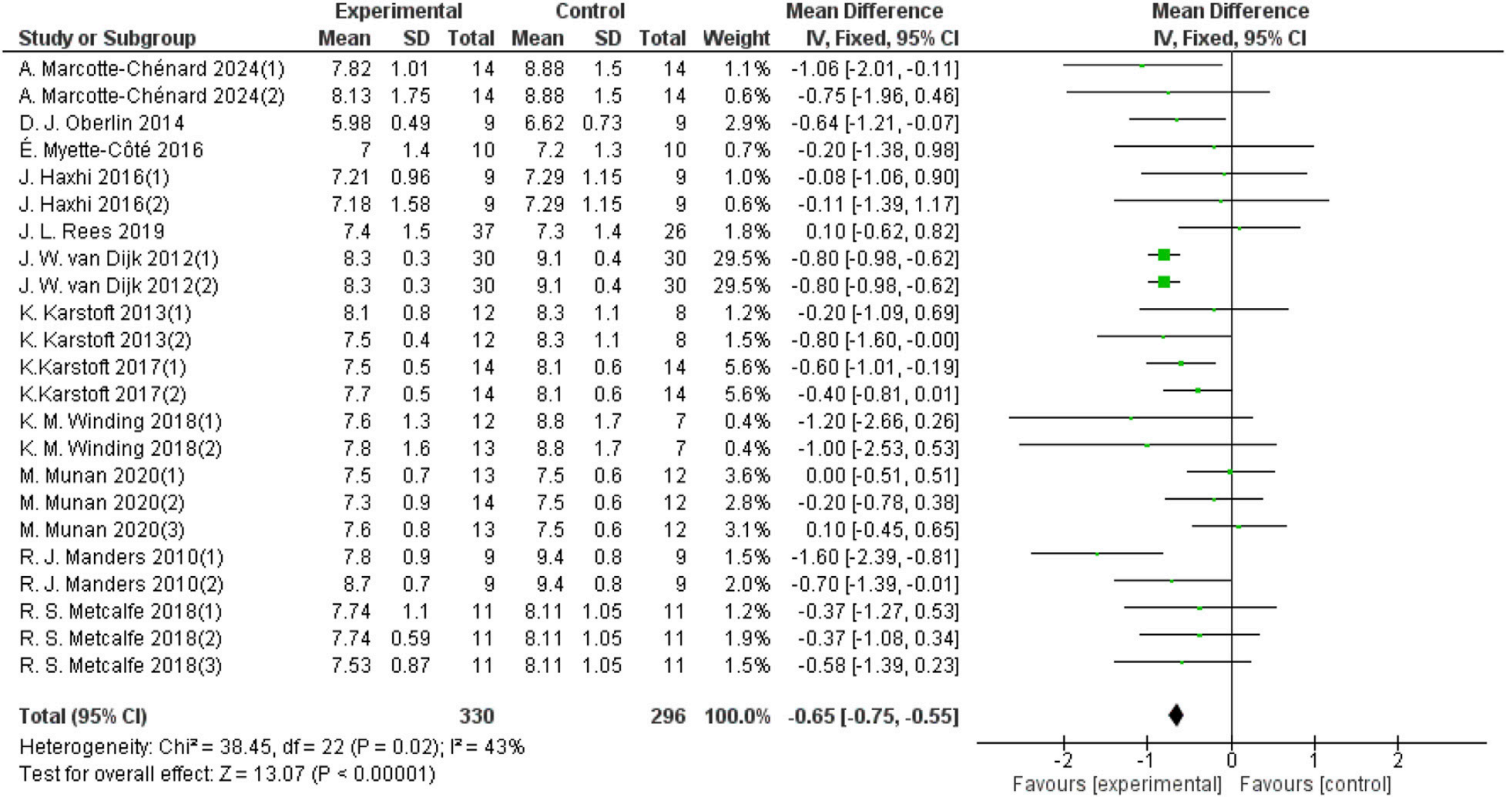
Forest plots showing the effect of aerobic exercise on 24-hour mean blood glucose in patients with T2DM.

Heterogeneity testing was performed on the included studies, and a fixed-effect model was used for meta-analysis. A total of 626 participants from 23 studies provided 24-hour mean blood glucose data. The results indicated that aerobic exercise can improve the 24-hour mean blood glucose in patients with T2DM (d = −0.65, 95% CI: −0.75 to −0.55, *p* < 0.05) (Figure 3).

**FIGURE 4:**
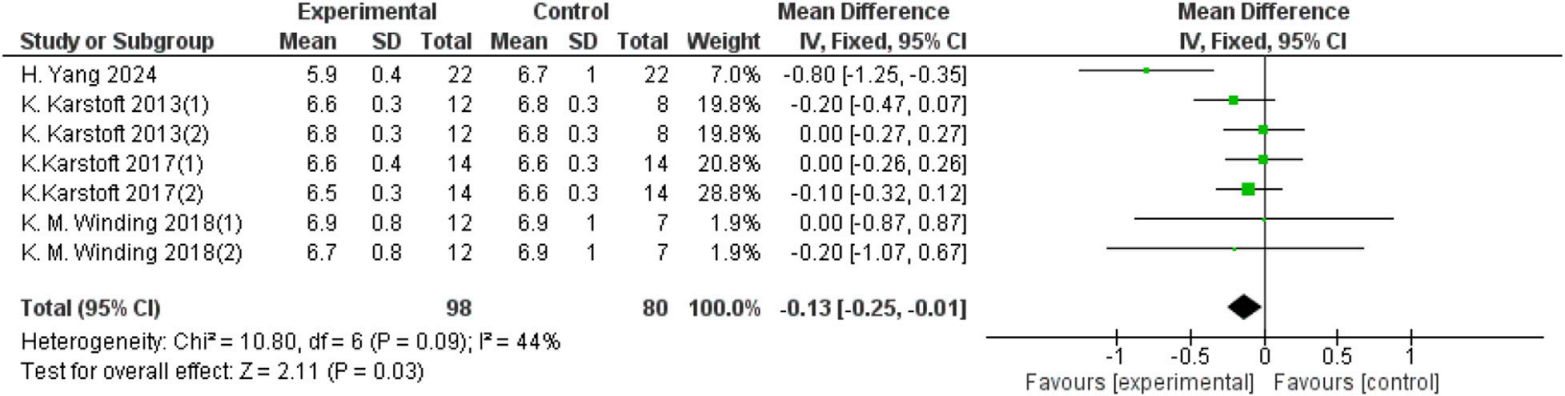
Forest plot showing the effect of aerobic exercise on HbA1c in patients with T2DM.

Heterogeneity testing was performed on the included studies, and a fixed-effect model was used for meta-analysis. A total of 178 participants from 7 studies provided HbA1c data, and aerobic exercise was found to improve HbA1c in patients with T2DM (d = −0.13, 95% CI: −0.25 to −0.01, *p* < 0.05) (Figure 4).

### 3.5 Subgroup analysis

#### 3.5.1 Effects of different periods of aerobic exercise on 24-h mean blood glucose in patients with T2DM

**FIGURE 5:**
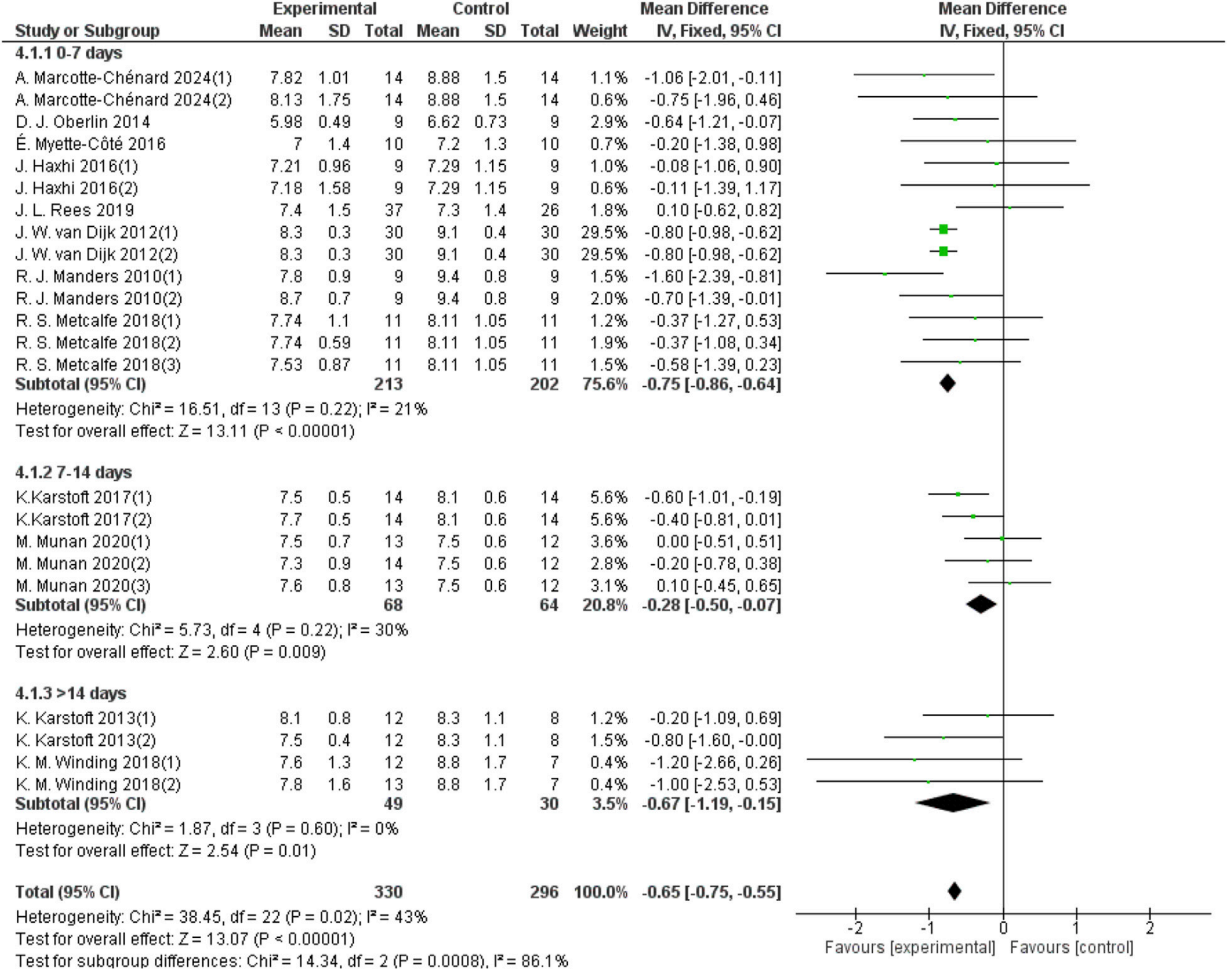
Forest plot of the effect of different periods of aerobic exercise on 24-hour mean blood glucose in patients with T2DM.

The aerobic exercise period was categorized into three groups: 0–7 days, 7–14 days, and more than 14 days. Fourteen studies with a total sample size of 415 provided data on 24-hour mean blood glucose for the 0–7-day aerobic exercise group. Five studies with a sample size of 132 reported data for the 7–14 days group, and four studies with a sample size of 79 provided data for the group with aerobic exercise lasting more than 14 days. As shown in Figure 5, aerobic exercise for 0–7 days significantly improved the 24-hour mean blood glucose in patients with T2DM (d = −0.75, 95% CI: −0.86 to −0.64, *p* < 0.05). Aerobic exercise for 7–14 days also led to a significant improvement (d = −0.28, 95% CI: −0.50 to −0.07, *p* < 0.05). Additionally, aerobic exercise for more than 14 days improved the 24-hour mean blood glucose (d = −0.67, 95% CI: −1.19 to −0.15, *p* < 0.05).

#### 3.5.2 Effect of different intensities of aerobic exercise on 24-h mean blood glucose in patients with T2DM

**FIGURE 6:**
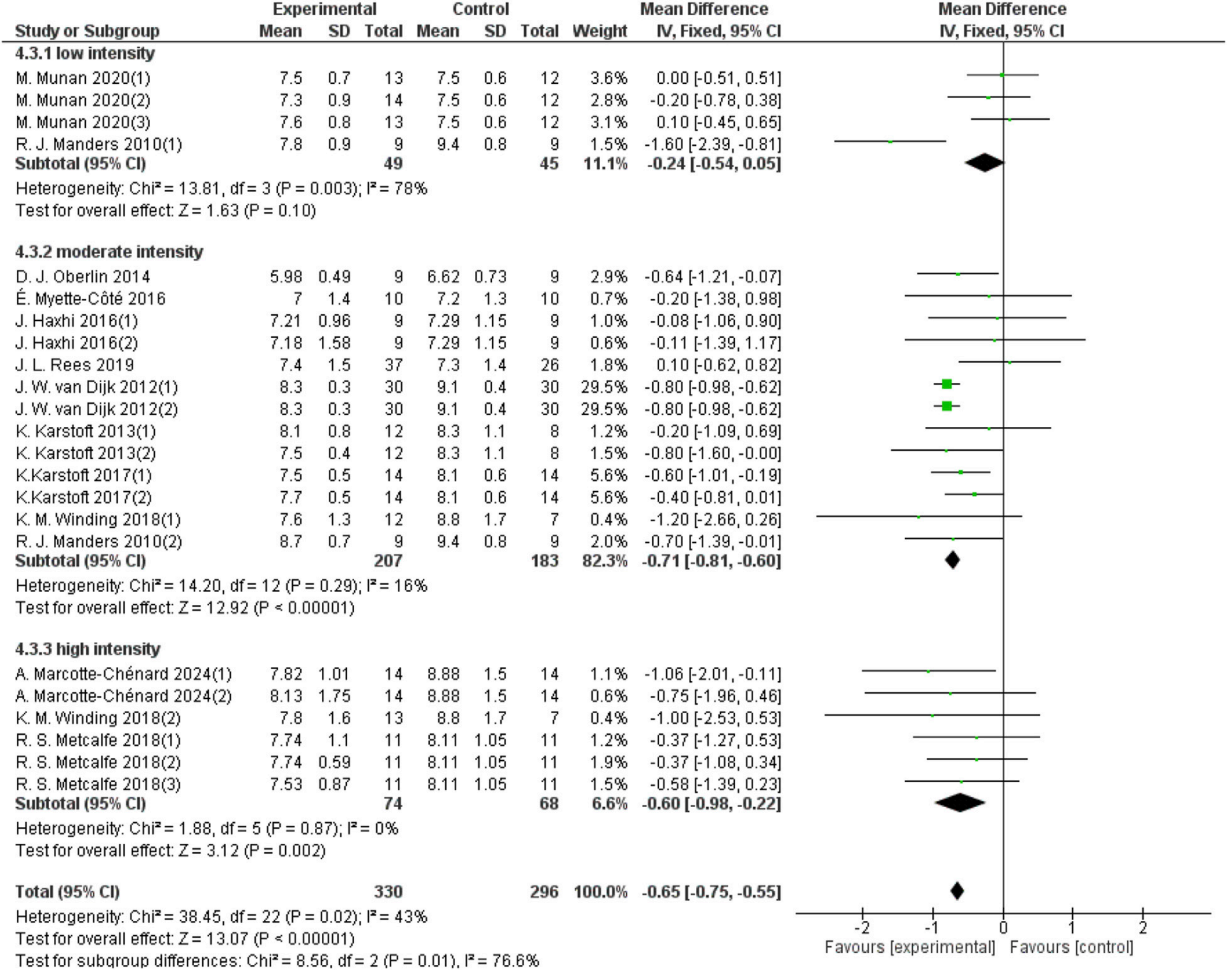
Forest plot showing the effect of different intensities of aerobic exercise on 24-hour mean blood glucose in patients with T2DM.

Aerobic exercise intensity was categorized into three groups: low-intensity, moderate-intensity, and high-intensity. Four studies with a total sample size of 94 in the low-intensity aerobic exercise group provided 24-hour mean blood glucose data; 13 studies with a sample size of 390 in the moderate-intensity aerobic exercise group provided 24-hour mean blood glucose data; and 6 studies with a sample size of 142 in the high-intensity aerobic exercise group provided 24-hour mean blood glucose data. As shown in Figure 6, low-intensity aerobic exercise cannot improve the 24-hour mean blood glucose of patients with T2DM (d = −0.24, 95% CI: −0.54 to 0.05, *p* < 0.05). Moderate-intensity aerobic exercise can improve the 24-hour mean blood glucose of patients with T2DM (d = −0.71, 95% CI: −0.81 to −0.60, *p* < 0.05). High-intensity aerobic exercise can improve the 24-hour mean blood glucose of patients with T2DM (d = −0.60, 95% CI: −0.98 to −0.22, *p* < 0.05).

#### 3.5.3 Effect of different frequencies of aerobic exercise on 24-h mean blood glucose in patients with T2DM

**FIGURE 7:**
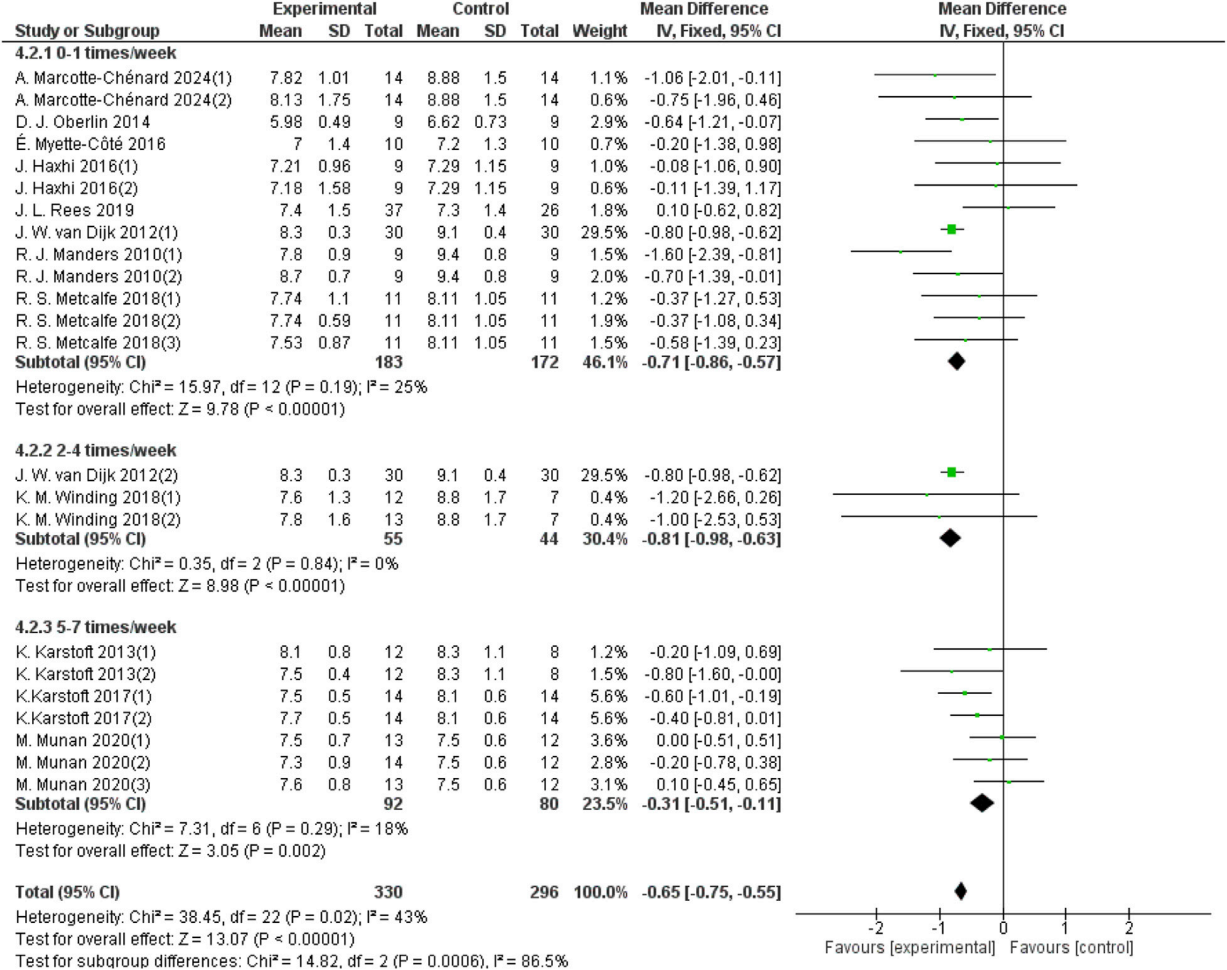
Forest plot showing the effect of different frequencies of aerobic exercise on 24-hour mean blood glucose in patients with T2DM.

The frequency of aerobic exercise was categorized into three groups: once per week, 2–4 times per week, and 5–7 times per week. Thirteen studies with a total sample size of 355 provided data on 24-hour mean blood glucose for aerobic exercise performed once a week; three studies with a sample size of 99 provided data for exercise 2–4 times per week; and seven studies with a sample size of 172 provided data for exercise 5–7 times per week. As shown in Figure 7, aerobic exercise performed once a week significantly improved the 24-hour mean blood glucose levels in patients with T2DM (d = −0.71, 95% CI: −0.86 to −0.57, *p* < 0.05). Aerobic exercise performed 2–4 times a week also showed a significant improvement (d = −0.81, 95% CI: −0.98 to −0.63, *p* < 0.05). The group of aerobic exercises performed 5–7 times a week improved the 24-hour mean blood glucose in patients with T2DM (d = −0.31, 95% CI: −0.51 to −0.11, *p* < 0.05).

#### 3.5.4 Effect of different durations of each aerobic exercise on 24-hour mean blood glucose in patients with T2DM

**FIGURE 8:**
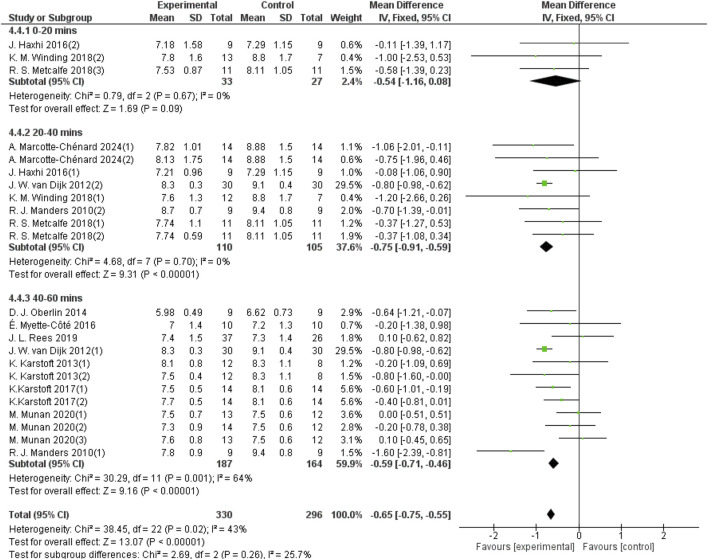
Forest plot showing the effect of different durations of aerobic exercise on 24-hour mean blood glucose in patients with T2DM.

The duration of aerobic exercise sessions was categorized into three groups: 0–20 min per session, 20–40 min per session, and 40–60 min per session. For the 20–40 min group, 8 studies provided data from a sample size of 215, and for the 40–60 min group, 12 studies provided data from a sample size of 351. As shown in Figure 8, aerobic exercise of 0–20 min per session did not significantly improve the 24-hour mean blood glucose levels in patients with T2DM (d = −0.54, 95% CI: −1.16 to 0.08, *p* < 0.05). In contrast, aerobic exercise performed for 20–40 min per session significantly improved the 24-h mean blood glucose (d = −0.75, 95% CI: −0.91 to −0.59, *p* < 0.05). Aerobic exercise for 40–60 min per session improved the 24-h average blood glucose in patients with T2DM (d = −0.59, 95% CI: −0.71 to −0.46, *p* < 0.05).

#### 3.5.5 Effects of aerobic exercise on 24-hour mean blood glucose in patients with different BMIs and T2DM

**FIGURE 9:**
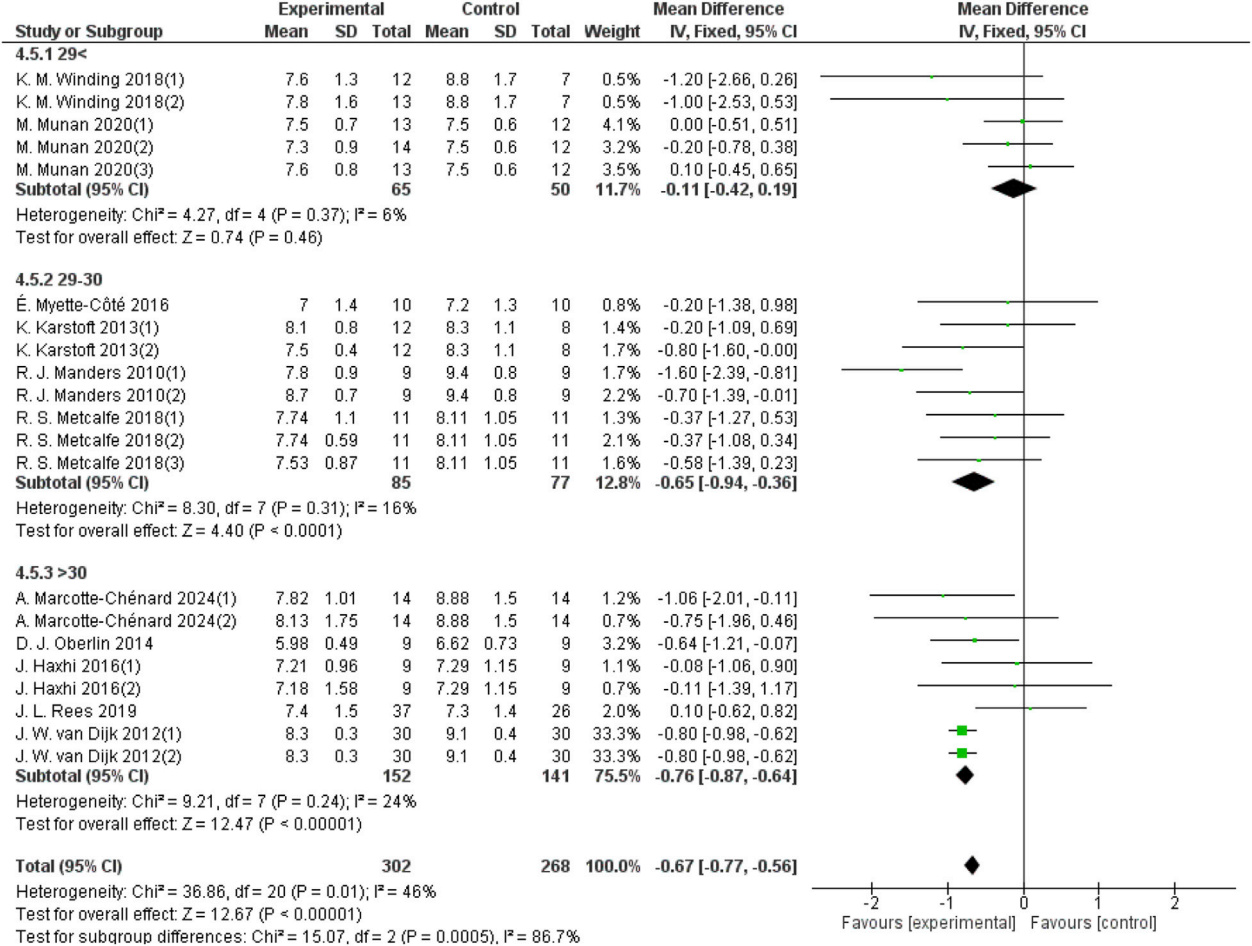
Forest plot showing the effect of aerobic exercise on 24-hour mean blood glucose in patients with T2DM and various BMIs.

Participants’ BMIs were divided into three groups: <29 kg/m^2^, 29–30 kg/m^2^, and >30 kg/m^2^. For the group with BMI <29 kg/m^2^, 5 studies provided 24-hour mean blood glucose data from a sample size of 115. For the 29–30 kg/m^2^ group, 8 studies provided 24-hour mean blood glucose data from a sample size of 162. For the BMI >30 kg/m^2^ group, 8 studies provided 24-hour blood glucose data from a sample size of 293. As shown in Figure 9, aerobic exercise did not significantly improve the 24-hour mean blood glucose in patients with T2DM in the BMI <29 group (d = −0.11, 95% CI: −0.42 to 0.19, *p* < 0.05). However, aerobic exercise significantly improved the 24-hour mean blood glucose levels in patients with a BMI between 29 and 30 (d = −0.65, 95% CI: −0.94 to −0.36, *p* < 0.05) and in those with a BMI >30 (d = −0.76, 95% CI: −0.87 to −0.64, *p* < 0.05).

### 3.6 Test for publication bias

**FIGURE 10:**
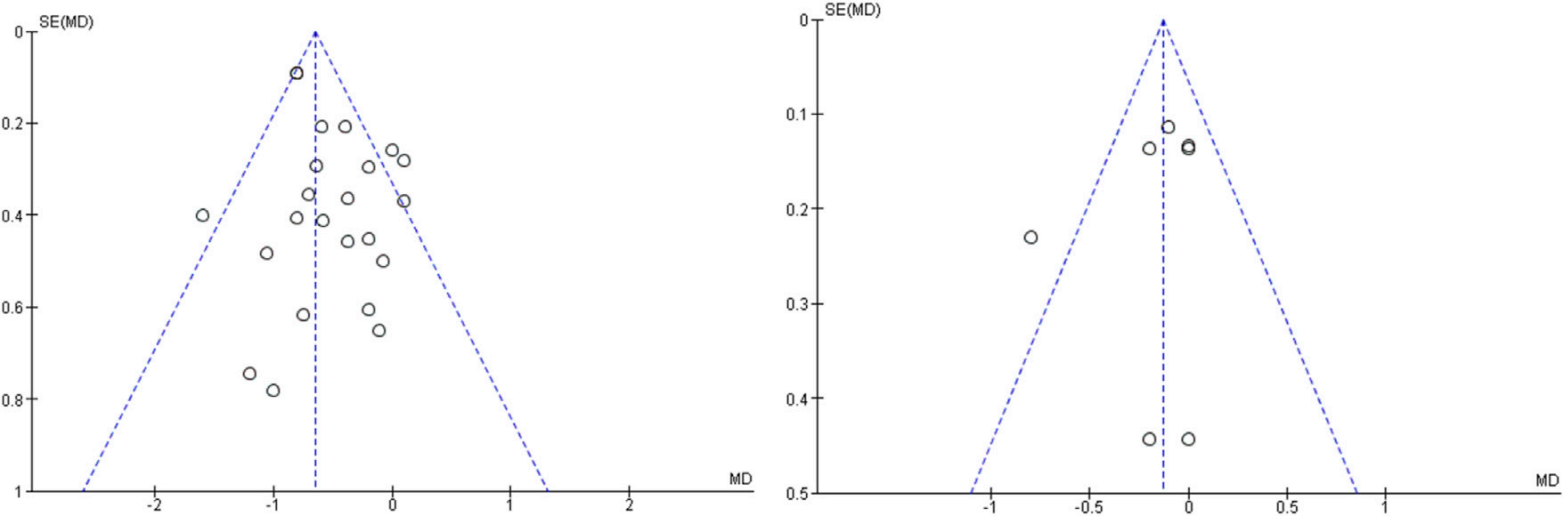
Publication bias funnel plot.

The outcome indicators of the included studies and the symmetrical distribution of the scatter points on both sides of the funnel plot suggest that there is no publication bias (see Figure 10).

### 3.7 Sensitivity analysis

A sensitivity analysis of the included studies, in which each article was excluded one by one until the improvement in the outcome indicators was not obvious, showed that the results of this study meta-analysis were reliable.

## 4 Discussion

### 4.1 Aerobic exercise can improve 24-hour mean blood glucose and HbA1c in patients with T2DM

This study found that aerobic exercise can improve 24-hour mean blood glucose and HbA1c in patients with T2DM, possibly by increasing muscle sensitivity to insulin. This is consistent with Al-Mhanna et al. (2024a), who found that combining aerobic and resistance training may increase muscle glucose uptake and promote favorable changes in body composition, such as reduced adiposity, thereby improving glycemic control and insulin sensitivity. Also, Al-Mhanna et al. (2023) found that combined aerobic exercise and diet significantly improved HbA1c concentration, fasting blood glucose, fasting plasma insulin, total cholesterol, triglycerides, C-reactive protein and series body composition in obesity and T2DM. Aerobic exercise activates multiple signaling pathways, especially AMP-activated protein kinase (AMPK) and protein kinase B (Akt), which promote the transport of glucose transporter 4 (GLUT4) from the bloodstream to the muscle cell membrane. This enhances muscle sensitivity to insulin, increasing the muscle’s ability to absorb and utilize glucose from the bloodstream, and reducing blood glucose levels (Dastbarhagh et al., 2019). Long-term aerobic exercise can sustain this effect, as reflected in a decrease in HbA1c (Qian et al., 2023).

### 4.2 Different periods of aerobic exercise can improve the 24-hour average blood glucose of patients with T2DM

This study concluded that different aerobic exercise periods can improve the 24-hour mean blood glucose in patients with T2DM. The most significant improvement was observed in the 0–7-day group, followed by the >14-day group, and lastly the 7–14-day group. A prolonged lack of exercise can reduce lipoprotein lipase activity, muscle glucose, and protein transporter activity, impair lipid metabolism, and decrease carbohydrate metabolism (Park et al., 2020). During the initial 0–7 days, participants without a regular exercise habit have not yet adapted to the physiological stimuli induced by exercise, resulting in a rapid increase in insulin sensitivity, with the most pronounced physiological response occurring during this period. As noted by Marcotte-Chénard et al. (2024), a single aerobic exercise session can significantly improve 24-hour mean blood glucose, blood glucose variability, and blood glucose peaks in patients with T2DM.

However, as the duration of exercise is extended to 14 days, the body gradually adapts to the exercise load, meaning it no longer needs to make drastic adjustments to cope with the same intensity of movement. This adaptation slows the improvement in insulin sensitivity, reducing the impact of exercise on the body. Timmons et al. (2005) found that 6 weeks of high-intensity aerobic circuit training significantly increased the mRNA expression levels of platelet reactant protein 4 and α2-macroglobulin in sedentary male participants, activated the expression of angiopoietin-1, tyrosine kinase with immunoglobulin-like and EGF-like domains 2 genes, and muscle extracellular matrix genes, promoted angiogenesis and vascular stability, improved muscle structure and function, and enhanced aerobic fitness and exercise adaptability. This adaptation process allows the body to handle the same exercise intensity more efficiently. Consequently, compared to the rapid changes observed in the first 7 days, the increase in insulin sensitivity tends to level off, making the exercise effect less pronounced than in the 0–7 days group.

After more than 14 days of continuous aerobic exercise, long-term aerobic exercise can improve fat oxidation and insulin sensitivity, allowing the body to utilize more fat as an energy source during exercise, thereby improving exercise outcomes. This is consistent with van Wouwe et al. (2018), who observed that a 10-month circuit type integrated neuromuscular training reduced body mass 6%, body fat 5.5%, and increased fat-free mass by 1.2%–3.4%, strength 27.2% and endurance 26.8% in obese women. Also long term high-intensity hybrid-type neuromuscular training and home-based circuit training efficiently decreased waist-to-hip ratio by 4.6% and total cholesterol by 26.5%, while improving cardiometabolic health (Alexios et al., 2021; Al-Mhanna et al., 2024b; Badri Al-mhanna et al., 2024). Similarly, Mendham et al. (2015) found that 12 weeks of exercise and gym workouts can significantly reduce the body mass index, waist circumference, and waist-to-hip ratio of male patients with T2DM, as well as leptin levels. Compared to the control group, the area under the insulin curve and insulin resistance was also reduced, while insulin sensitivity increased. These findings suggest that continuous aerobic exercise for more than 14 days yields superior metabolic and glycemic control compared to shorter durations (7–14 days).

### 4.3 Different intensities of aerobic exercise can improve 24-hour mean blood glucose in patients with T2DM

This study concluded that low-intensity aerobic exercise cannot improve the 24-h mean blood glucose levels in patients with T2DM, whereas moderate- and high-intensity aerobic exercise can effectively reduce blood glucose levels, with moderate-intensity aerobic exercise being more effective than high-intensity aerobic exercise. Similarity, Badri et al. (2024) reported that moderate to high-intensity exercise protocols were highly efficient for progressively increasing HDL-C levels, reducing body mass, and lowering cardiovascular disease risk factors in obesity individuals. These findings highlight the importance of moderate and high exercise intensity in managing cardiometabolic health in individuals with obesity or type 2 diabetes. Low-intensity exercise generally consumes less energy and cannot decrease blood sugar and, therefore, has no significant effect on blood sugar regulation (Zhang et al., 2021). For example, Munan et al. (2020) found that a single morning fasting walk did not significantly improve the 24-hour mean blood glucose levels in patients with T2DM, and compared with the evening exercise group, the respiratory exchange rate was reduced, and compared with the afternoon exercise group, the blood glucose level was not significantly reduced.

Additionally, Batrakoulis et al. (2022b) suggested that an exercise intensity of 40%–60% of the oxygen consumption reserve is ideal for improving cardiometabolic health in patients with T2DM. Moderate-intensity exercise can moderately increase the body’s energy expenditure, promote glucose utilization, and improve insulin sensitivity so that the body can more effectively utilize blood sugar, thereby reducing the 24-hour average blood sugar level. For example, Motahari-Tabari et al. (2014) found that 8 weeks of moderate-intensity aerobic exercise can significantly reduce the weight, waist circumference, hip circumference, BMI, plasma insulin, and insulin resistance of female patients with T2DM and promote blood glucose control. For high-intensity exercise, it can significantly increase energy expenditure and metabolic rate, but it can also have drastic adverse effects, making it less efficient than moderate intensity. High-intensity exercise triggers an immediate physiological stress response, causing a temporary increase in the levels of catecholamines (such as adrenaline and noradrenaline) (Liu et al., 2020). These hormones acutely promote the breakdown of glycogen in the liver, increase blood glucose concentration, which may temporarily counteract the hypoglycemic effect of exercise (Carletti et al., 2023).

### 4.4 Different frequencies of aerobic exercise can improve 24-hour mean blood glucose in patients with T2DM

This study found that aerobic exercise at different frequencies can improve the 24-hour mean blood glucose levels in patients with T2DM. The most effective exercise frequency is 2–4 times per week, followed by once per week, with 5–7 times per week having the least effect. The group of aerobic exercises 2–4 times a week can continuously improve the body’s metabolic capacity during gradual adaptation, thereby achieving optimal blood glucose control. For example, Winding et al. (2018) found that 11 weeks of three-times-a-week HIIT can significantly reduce visceral fat mass, HbA1c, FBG, postprandial blood glucose, blood glucose variability, and HOMA-IR in patients with T2DM, and improve the overall health, body composition ratio and blood glucose control ability of patients. In contrast, exercising once per week primarily induces transient changes in blood glucose levels due to the lack of consistent metabolic stimulation and adaptation. Cerqueira et al. (2020) claimed that no significant changes were observed following either a single 45-minute session of moderate exercise or a 5-minute session of high-intensity exercise. Similarly, Haxhi et al. (2016) found that single session of moderate-intensity exercise only minimally decrease patients with T2DM 24-hour mean blood glucose from 7.29 ± 1.15 to 7.21 ± 0.96. Additionally, patients who exercise 5–7 times per week may experience stress responses due to overtraining, leading to increased levels of stress hormones (such as cortisol), which could negatively impact blood glucose control (Hackney and Lane, 2015). Karstoft et al. (2013) found that 4 months of moderate-intensity exercise performed five times per week resulted in only a slight decrease in 24-hour mean blood glucose (from 8.3 ± 1.1 to 8.1 ± 0.8) in patients with T2DM. This suggests that excessively high exercise frequency may not provide additional benefits and could even be less effective than moderate frequencies.

### 4.5 Different durations of each aerobic exercise can improve the 24-hour mean blood glucose in patients with T2DM

This study found that aerobic exercise lasting less than 20 min cannot improve the 24-hour mean blood glucose of T2DM patients. In comparison, 20–40 min and 40–60 min of aerobic exercise can improve the 24-hour mean blood glucose level in patients with T2DM, with the 20–40-minute duration proving to be the most effective. When the duration of exercise is less than 20 min, exercise may not be able to fully activate the uptake and utilization of glucose in muscles and thus fail to change blood glucose levels throughout the day significantly. For example, Metcalfe et al. (2018) found that a single 10 × 1-minute HIIT can significantly reduce the duration of hyperglycemia in patients with type 2 diabetes, while blood glucose variability does not significantly change from the 24-hour average blood glucose. Second, 20–40 min may be the best combination of intensity and time. Sufficient time allows the body’s energy metabolism to be fully activated, improving oxidative stress while not causing excessive stress or fatigue due to excessive duration. For example, Haxhi et al. (2016) found that 40 min of moderate-intensity aerobic exercise significantly reduced the duration of hyperglycemia and urinary isoprostanes, alleviated oxidative stress, and lowered the 24-hour mean blood glucose. Similarity, On the other hand, prolonged exercise durations of 40–60 min may lead to excessive fatigue, potentially hindering long-term metabolic improvements (Liu et al., 2024).

### 4.6 Aerobic exercise can improve the 24-hour mean blood glucose in patients with T2DM and various BMIs

This study concluded that aerobic exercise cannot improve the 24-hour mean blood glucose in patients with T2DM who have a BMI below 29 kg/m^2^. In contrast, aerobic exercise can improve these levels in patients with a BMI between 29 and 30 kg/m^2^, and those with a BMI above 30 kg/m^2^, with the most significant effect observed in patients with a BMI above 30 kg/m^2^. A BMI below 29 kg/m^2^ generally indicates a lower body fat percentage; however, individuals with this BMI may still experience insulin resistance or other metabolic issues, which are not solely related to body fat. Consequently, their response to exercise in terms of blood glucose control may be less pronounced (Butryn et al., 2019). Patients with a BMI between 29k and 30 kg/m^2^, and those with a BMI above 30 kg/m^2^, often face greater challenges in managing self-monitoring, inhibitory control, and planning abilities. Despite the substantial effort required, the benefits of exercise, including fat loss and overall metabolic improvement, are typically more pronounced in these groups. Batrakoulis (2022a) and Batrakoulis (2022b) found that yoga activity and Pilates Training can efficiently reduce reductions in body weight, BMI, body fat, waist size, waist-to-hip ratio and lipid metabolism in overweight and obese individuals. This is likely due to the greater impact of exercise on body composition and metabolic function in individuals with higher BMIs, leading to more significant improvements in blood glucose control (Butryn et al., 2019).

### 4.7 Limitations

This study is the first meta-analysis to analyze the effect of aerobic exercise on 24-hour mean blood glucose in patients with T2DM as measured by CGM. It provides insights into the overall effect of aerobic exercise on 24-hour mean blood glucose and HbA1c in T2DM patients, as well as a detailed subgroup analysis of how different exercise periods, intensities, frequencies, and durations affect patients with varying BMIs. However, this meta-analysis still has some limitations. First, the studies we included were randomized controlled trials (RCTs) with relatively small sample sizes, which resulted in an underrepresented patient population and may have introduced selection bias. More high-quality RCTs are needed to explore their effectiveness in the future. Secondly, since diabetes and its complications are chronic diseases, most current research is based on short- and medium-term studies, and there is a lack of research data on long-term interventions. Therefore, future research should focus on long-term interventions.

## 5 Conclusion

5.1. Aerobic exercise can improve 24-hour mean blood glucose and HbA1c in patients with T2DM.
5.2. Aerobic exercise for 0–7 days, 7–14 days, and >14 days can improve the 24-hour mean blood glucose in patients with T2DM.
5.3. Moderate- to high-intensity aerobic exercise can improve the 24-hour mean blood glucose level in patients with T2DM patients, whereas low-intensity exercise cannot.
5.4. Aerobic exercise once a week, as well as 2–4 times and 5–7 times per week, can improve the 24-hour mean blood glucose of T2DM patients.
5.5. Each 20–40 and 40–60 min of aerobic exercise can improve the 24-hour mean blood glucose in patients with T2DM, whereas the 0–20 min of aerobic exercise cannot improve it.
5.6. Aerobic exercise can improve the 24-hour mean blood glucose in patients with T2DM who have a BMI between 29 and 30 and above 30 kg/m^2^ but not in those with a BMI below 29.

## Data Availability

The original contributions presented in the study are included in the article/supplementary material, further inquiries can be directed to the corresponding author.
